# Electromyographic activity of quadriceps muscle during sit-to-stand in patients with unilateral knee osteoarthritis

**DOI:** 10.1186/s13104-018-3464-9

**Published:** 2018-06-05

**Authors:** Hamad S. Al Amer, Mohamed A. Sabbahi, Hesham N. Alrowayeh, William J. Bryan, Sharon L. Olson

**Affiliations:** 1 Department of Physical Therapy, Faculty of Applied Medical Sciences, University of Tabuk, Tabuk, 71491 Saudi Arabia; 2 School of Physical Therapy, Texas Woman’s University, 6700 Fannin Street, Houston, TX 77030 USA; 3 Physical Therapy Department, Faculty of Allied Health Sciences, Kuwait University, 90805 Sulaibekhat, Kuwait; 4 Department of Orthopedics, The Methodist Hospital, 6565 Fannin Street, Houston, TX 77030 USA

**Keywords:** Knee, Osteoarthritis, Quadriceps muscle, Sit-to-stand, Electromyography

## Abstract

**Objective:**

The sit-to-stand (STS) is a simple test to evaluate the functional performance of the quadriceps muscle in patients with knee osteoarthritis (OA). The aim was to evaluate the electromyographic (EMG) activity of the ipsilateral quadriceps during STS task at different seat heights and feet positions in patients with severe unilateral OA. The EMG activity was recorded in a group of eight participants with unilateral OA during the performance of STS task in four conditions: (1) knee-height seat with feet together, (2) knee-height seat with feet askew (feet side by side and heel-to-toe), (3) low-height seat (25% lower than knee-height seat) with feet together, and (4) low-height seat with feet askew.

**Results:**

There was a statistically significant difference among the four conditions in the EMG activity (*p *=0.004). Particularly, the EMG activity of the quadriceps was significantly higher when participants rose from the low height with their feet askew than when they rose from the knee height with their feet placed together (*p *=0.004) or askew (*p *=0.002). These results recommend considering initial feet position and seat height when evaluating the functional activity of the quadriceps in patients with unilateral OA using STS test.

## Introduction

The knee joint is the joint most commonly affected by osteoarthritis (OA) [1]. Patients with knee OA usually suffer from pain, limited range of motion, stiffness and muscle weakness [2]. Therefore, knee OA has been recognized as a major source of disability and physical impairment in older adults [3].

Quadriceps muscle weakness is a common clinical feature of knee OA [4–6]. Persistent weakness of the quadriceps plays a major role in increasing the stress over the knee joint and progression of joint damage [7]. Hence, improving the functional strength of the quadriceps in patients with knee OA has received great attention in the literature [8–10].

The assessment of the quadriceps muscle is warranted to determine the functional status of patients with OA. The sit-to-stand (STS) test is a performance-based measure frequently used in patients with knee OA to measure the functional performance of the quadriceps muscle. Sufficient quadriceps force is required to complete the STS movement. Therefore, quadriceps weakness was found to have a significant impact on STS performance [11–13].

Electromyography (EMG) is commonly used to obtain information about the effects of chair seat height and initial feet positions on the activity of the lower limb muscles during STS movement [14–17]. Measuring EMG activity of the knee extensors during these tasks would reflect the amount of loading applied to the quadriceps. This is a key muscle to be targeted during the rehabilitation program of patients with knee OA. Therefore, the purpose of this study was to evaluate the EMG activity of the quadriceps muscle, specifically the vastus lateralis (VL), during STS task at different seat heights and feet positions in individuals with severe unilateral OA. The data presented in this study is a side product of another unpublished research project investigating the activity of thigh musculatures during selected functional activities before and after total knee arthroplasty (TKA).

## Main text

### Methods

Eight participants (five males and three females) volunteered for the study 1–2 weeks before undergoing unilateral elective TKA. The mean age of the participants was 64.61 ± 11.01 years and the body mass index was 34.06 ± 8.89 kg/m^2^. Participants were included based on the following criteria: no other musculoskeletal disorders or neurological pathologies; and no previous hip, knee, spine or neck surgery within the past year.

EMG activity was sampled at 1000 Hz and sweep speed of 100 points/s. using the Myosystem 1200 version 2.11 (Noraxon USA, Inc., Scottsdale, AZ) via the Telemyo 900 telemetry unit (Noraxon USA, Inc., Scottsdale, AZ). Two adhesive surface electrodes were placed over the mid-muscle belly of the VL of the arthritic knee. The electrodes were placed longitudinally in a bipolar configuration with inter-electrode distance of 2 cm. A ground electrode was affixed over the fibular head. The VL was chosen in this study as a representative of the quadriceps muscles based on its several unique characteristics. The VL is considered the largest among the four quadriceps muscles [18] and the main generator of extension torque at the knee [19]. Although there is no difference in time of onset among the four muscles of the quadriceps during closed-chain movements, the VL has the largest amount of EMG activity during that type of movement [20].

**Fig. 1:**
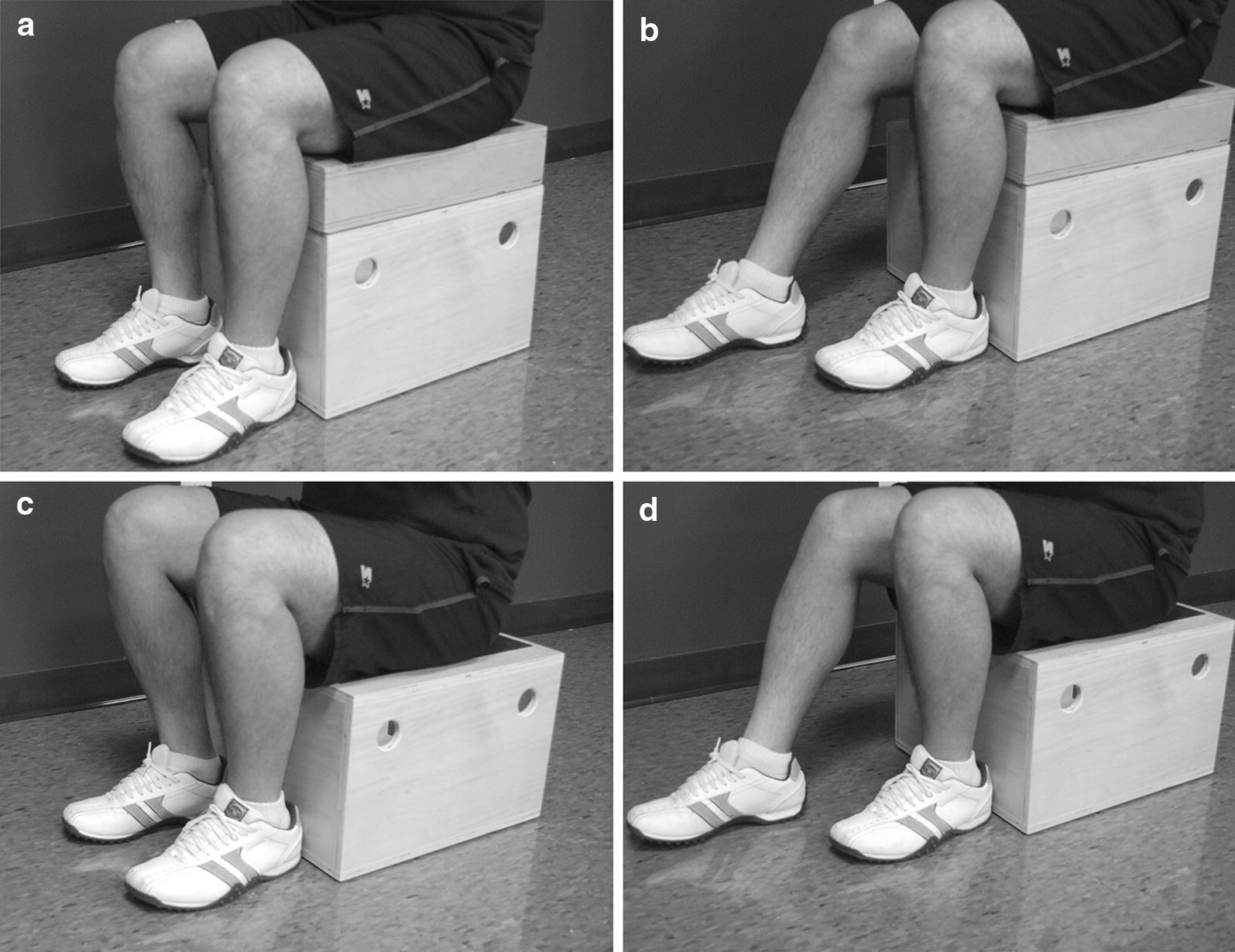
The four conditions of sit-to-stand task. **a** Normal height feet together (NHFT). **b** Normal height feet askew (NHFA). **c** Low height feet together (LHFT). **d** Low height feet askew (LHFA)

The participants performed STS tasks in the following order: STS at normal height (knee-height seat) with feet together (Fig. 1a), STS at normal height with feet askew (feet side by side, heel-to-toe with foot of arthritic knee behind the other) (Fig. 1b), STS at low height (25% lower than knee-height seat) with feet together (Fig. 1c), and STS at low height with feet askew (Fig. 1d) (*tasks hereafter will be identified as NHFT, NHFA, LHFT and LHFA, respectively*). The starting position was sitting on an armless, backless chair, and maintaining feet flat on the floor and thighs at hip width. To perform the tasks, participants were instructed to stand while holding arms across the chest with weight equally distributed on both feet in NHFT and LHFT. In NHFA and LHFA, they had the chance to load their feet as they wish to complete the task. Two trials of each STS task were performed and used for analysis.

For normalizing the EMG data, participants completed two maximal voluntary isometric contractions (MVIC) using an electromechanical dynamometer (Biodex Inc., Shirley, NY) while EMG was recorded. The examined limb was stabilized with the hip and knee flexed to 90° and 15°, respectively. Two 10-s trials were recorded with 2 min of rest in between.

To analyze EMG signals of quadriceps, raw signals were full-wave rectified and smoothed with a 10 ms window. The obtained linear envelope was analyzed to determine the peak EMG signal amplitudes during the STS task trials and during MVIC trials. Because it is common to find high levels of EMG activity during dynamic tasks versus MVIC [13], the two trials of each STS task were also examined for the maximum EMG signal. Subsequently, the peak EMG signal obtained during the STS tasks trials was normalized to the maximum EMG signal obtained either during the MVIC or during the STS trials, whichever had higher activity. This normalization method is common in EMG studies [13, 21, 22] and was suggested as a more accurate method since all the normalized peak EMG activity would fall below or equal to 100% of the maximum signal [13]. The normalized peak EMG activity of VL during the two trials of each task was averaged and used as the final outcome score for the performed task.

Statistical analysis included one independent variable: the STS task, with four levels (NHFT, NHFA, LHFT and LHFA). The dependent variable was the normalized peak EMG activity of the quadriceps muscle measured in percent of maximum activation. A one-way analysis of variance (ANOVA) for repeated measures (univariate approach) was conducted to test the main effect of the independent variable with alpha level set at 0.05. The univariate approach was selected due to the small sample size. Regarding the sphericity assumption, Geisser-Greenhouse epsilon hat ($\hat{\varepsilon }$) of 0.764 was found. Therefore, the degrees of freedom (factor and error) were adjusted according to this value to prevent inflation of alpha.

### Results

**Fig. 2:**
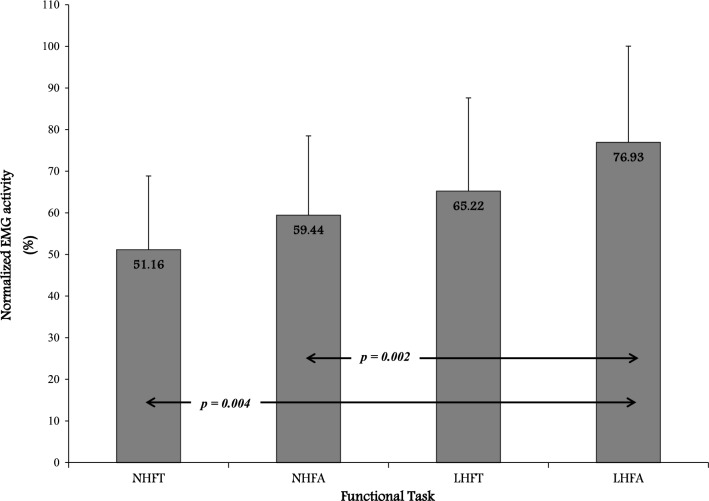
The means and standard deviations in each condition. NHFT: normal height feet together, NHFA: normal height feet askew, LHFT: low height feet together, LHFA: low height feet askew

The means and standard deviations of the normalized EMG for each condition are illustrated in Fig. 2. The result of the one-way ANOVA showed a statistically significant difference among the four conditions in the normalized EMG activity, *F* (2.29, 16.04)=7.54, *p *=0.004.

All pairwise comparisons were conducted to examine the difference among tasks using Bonferroni tests. Alpha level was adjusted to .05/6 (number of comparisons) = 0.0083 to prevent type I error. All pairwise comparisons revealed the normalized EMG activity was significantly higher in LHFA (M = 76.93%) than in NHFT (M = 51.16%) or NHFA (M = 59.44%) (Fig. 2). No further differences were found among the rest of conditions. Table 1 displays the *t*-statistic, degrees of freedom and *p* value for each comparison.

**Table 1:** Results of all pairwise comparisons for the normalized EMG during different sit-to-stand tasks

Pairwise comparison	*t*	*df*	*p*-*value*
NHFT vs. NHFA	1.47	7	0.186
NHFT vs. LHFT	2.62	7	0.035
NHFT vs. LHFA	4.25	7	0.004^a^
NHFA vs. LHFT	1.09	7	0.311
NHFA vs. LHFA	4.63	7	0.002^a^
LHFT vs. LHFA	1.71	7	0.130

*NHFT* normal height feet together, *NHFA* normal height feet askew, *LHFT* low height feet together, *LHFA* low height feet askew

^a^Indicates significant difference at α = 0.0083

### Discussion

This study was conducted to evaluate the effect of four different conditions of STS task on the EMG activity of the quadriceps muscle in individuals with severe unilateral OA. The findings showed that during STS movement, the activity of the quadriceps is modulated by the chair height and feet position.

A possible explanation of the difference in EMG activity of the quadriceps between the LHFA and NHFT positions is that the participants could be trying to avoid loading the arthritic knee during the latter due to possible weakness of the quadriceps muscle, pain, or both. When the arthritic and unaffected sides’ feet were parallel, participants had the chance to compensate for the arthritic side which might be weaker than the unaffected side. This phenomenon has been observed in patients with unilateral TKA [12, 13]. Farquhar et al. [12]. found the activity of the quadriceps muscle on the involved side to be significantly lower than the uninvolved side during STS task up to 3 months following the surgery. Due to significant weakness in the knee extensors, patients avoided loading of the involved limb by shifting the load to the uninvolved limb. However, because the EMG activity of the quadriceps at the uninvolved side was not recorded in the present study, we are not sure if the participants used the same compensatory approach during the performance of the STS task. Nevertheless, quadriceps weakness is a common feature in patients with knee OA and patients with TKA in the early phases following the surgery [13].

Repositioning the foot of the unaffected side anterior to the foot of the arthritic side significantly increased the activity of the quadriceps. Generally, placing the feet posteriorly moves the ground reaction force vector further posteriorly with respect to the knee, leading to a higher external flexion moment applied on that knee [23]. In this study, the relatively posterior position of the arthritic knee’s foot produced higher demand on the ipsilateral quadriceps to overcome the increase in the ground reaction force. Additionally, this position retained the arthritic knee closer to the center of gravity [24]. As a result, the arthritic knee was the principal leg to perform the upward displacement of the body. For that reason, the relatively posterior position of the foot of the arthritic knee required the participants to use that knee instead of the unaffected one as compensation, due to possible weakness or pain avoidance of the affected side.

The reported increase in the EMG activity of the quadriceps during STS movement from a low height in comparison to those with knee height (with feet askew in both tasks) suggests increasing the demand on the knee extensors. This finding is in agreement with previous research [14, 15, 25]. Arborelius et al. [15] examined the effect of rising from two different seat heights in healthy individuals and found a significant increase in the activity of the VL muscle with rising from a lower seat height in comparison to higher seat height. As the seat height decreases, the knee flexion angle and the knee flexion moment will increase. This would lead to higher demand on the quadriceps muscle to extend the knee in lifting the body weight [15, 26].

Performing the STS test with placing both feet together provides an opportunity for the patients to use the uninvolved side to compensate for the possible weakness of the arthritic side. This may not reflect the true status of the quadriceps performance on the affected side. Conversely, repositioning the unaffected side anterior to the arthritic side imposes more demand on the patient to use the involved side instead of compensating with the uninvolved side. This task better demonstrates the true functional performance of the ipsilateral quadriceps muscle. Furthermore, lowering the seat height will add greater difficulty to the test as it places more demand on the side being tested. Therefore, starting positions with regard to chair height and initial feet position need to be standardized in order to avoid misleading results.

To conclude, the results of this study indicate that the modification of seat height and feet position during STS movement plays an important role in clinically evaluating patients with knee OA. Lowering the seat height and placing the foot of the unaffected side anterior to the foot of the arthritic side increase the demand on the quadriceps muscle of the arthritic knee. This starting position would prevent patients from utilizing some strategies to avoid using their arthritic side to complete the STS task. This, in turn, may reflect the true functional condition of the knee extensors in patients with knee OA and the potential need of additional intervention.

## Limitations

A limitation of this study is the small sample size, which may have affected the significance of the results. Particularly when Bonferroni adjustment was used. Another potential source of type II error is the high variability of the EMG data. In fact, some electrophysiological studies used a liberal level of significance when analyzing EMG data in order to avoid type II error e.g. [12, 13, 21, 22]. Another limitation is the lack of EMG testing for the unaffected limb, and lack of quadriceps strength measurements. Those recordings could have supported the study’s findings.

## Abbreviations

OA: Osteoarthritis
STS: Sit-to-stand
EMG: Electromyography
VL: Vastus lateralis
TKA: Total knee arthroplasty
NHFT: Normal height with feet together
NHFA: Normal height with feet askew
LHFT: Low height with feet together
LHFA: Low height with feet askew
MVIC: Maximal voluntary isometric contraction
ANOVA: Analysis of variance
